# A qualitative assessment of perspectives on getting pregnant: the Social Position and Family Formation study

**DOI:** 10.1186/s12978-019-0793-7

**Published:** 2019-09-05

**Authors:** Meredith G. Manze, Dana Watnick, Diana Romero

**Affiliations:** 1 Graduate School of Public Health and Health Policy, City University of New York (CUNY), 55 W. 125th St., New York, NY 10027 USA; 2 Center for AIDS Research, Albert Einstein College of Medicine, New York, NY USA

**Keywords:** Pregnancy intention, Qualitative research

## Abstract

**Background:**

Intentions-oriented approaches to measuring pregnancy do not necessarily align with how people view and approach pregnancy. Our objective was to obtain an in-depth understanding of the notions women and men hold regarding pregnancy.

**Methods:**

We conducted semi-structured in-depth interviews with 176 heterosexual women and men ages 18–35, in the United States. Data were analyzed using grounded theory methodology.

**Results:**

Participants described notions of getting pregnant in one of three ways. One group of participants used language that solely described pregnancy as a deliberate process, either premeditated or actively avoided. Another described pregnancy as a predetermined phenomenon, due to fate or something that ‘just happens.’ The third group represented a blending of both notions.

**Conclusions:**

Our findings underscore the need to shift the current paradigm of deliberate *intentions* to one that recognizes that pregnancy can also be viewed as *predetermined*. These findings can be used to improve measurement, health services, and better direct public health resources.

## Plain English summary

Researchers and clinicians tend to measure perceptions around pregnancy by asking women if a pregnancy was intended or not. However, this is not necessarily how most people think about and approach pregnancy. Our objective was to obtain an in-depth understanding of the notions women and men hold regarding pregnancy. We interviewed 176 heterosexual women and men, ages 18–35 in the U.S. Participants described notions of getting pregnant in one of three ways. One group of participants used language that solely described pregnancy as a deliberate process, either premeditated or actively avoided. Another described pregnancy as a predetermined phenomenon, due to fate or something that ‘just happens.’ The third group represented a blending of both notions. Our findings underscore the need to shift how we think about and measure pregnancy, from one of just deliberate *intentions* to one that recognizes that pregnancy can also be viewed as *predetermined*. These findings can be used to improve measurement, health services, and better direct public health resources.

## Background

Researchers and clinicians have made numerous attempts over the last several decades to measure perceptions and experiences of becoming pregnant, primarily using an intentions-based framework. The result has been to operationalize the construct of ‘pregnancy intentions’ and to measure limited aspects of the phenomenon of becoming pregnant, namely if a pregnancy was wanted or timed according to an individual’s desires [1, 2]. Given this, the current measurement tools we use to conceptualize pregnancy over-simplifies classification of the phenomenon into “intended” and “unintended,” with unintended pregnancy defined as those that are mistimed or unwanted. A mistimed pregnancy is one that occurs earlier than desired, whereas an unwanted pregnancy is defined as occurring when a pregnancy is not desired [2].

Pregnancy intentions are primarily asked of women and assessed for three main purposes: 1) to estimate the prevalence, determinants, and consequences of intended and unintended pregnancies, 2) to use these estimates to address population-level needs such as access to contraception and abortion, and 3) to determine if/what reproductive health counseling should be delivered to a specific patient during a clinical encounter [2–4]. To estimate prevalence rates, pregnancy intentions are often measured in large-scale surveys using a series of questions such as “So would you say you became pregnant too soon, at about the right time, or later than you wanted?” [5] Researchers have noted shortcomings in these measurements, such as the possibility of a real change in attitudes pertaining to a previous pregnancy when data are collected retrospectively, as well as social desirability bias subsequent to the birth of a child [2, 6, 7]. Pregnancy intentions are increasingly being assessed prospectively during clinic visits [8]. Recent initiatives train clinicians to ask female patients of their intentions to become pregnant, and, depending upon the response, to initiate preconception care and/or contraceptive counseling [3, 9].

Pregnancy intention measures attempt to capture how the phenomenon of becoming pregnant is broadly conceptualized [10]. However, many of the commonly used measures employ language that does not necessarily align with how women view pregnancy [11–14]. Intentions-oriented approaches to pregnancy assume that active, prospective decision-making in becoming pregnant is universal [2, 15] and that all individuals have a plan with regard to childbearing. More recent measures have attempted to capture an expanded understanding of pregnancy intention by incorporating the notions of happiness or ambivalence related to pregnancy [6, 15–17]. This approach, however, remains limited in capturing the complex range of thoughts and beliefs people have about becoming pregnant [2, 6, 18, 19]. A more recent, comprehensive assessment has incorporated concepts such as partner discussions and feelings about motherhood, but maintains an intentions-based conceptualization of pregnancy and, thus far, has only been tested with women [20, 21].

According to the definition described above, unintended pregnancy in the U.S. can be considered at epidemic proportions, with an estimated 45% of all pregnancies meeting this definition in 2011 [22]. In response, social services and public health efforts have invested substantial resources towards reducing unintended pregnancies, oftentimes framing this as a problem with knowledge of, access to, or inconsistent use of contraception [23, 24]. Although increasing knowledge about and access to contraception is clearly beneficial, the disconnect between pregnancy intentions measurement and how some people approach pregnancy suggests that an over-emphasis on (reducing) unintended pregnancies may be misguided. Recognizing this, it is prudent to question the underlying theoretical assumption embedded into a pregnancy intentions framework that it is instinctive and ideal for all people to plan their pregnancies.

While some studies suggest that there is a causal association between lack of pregnancy planning and poor health outcomes for the parents and child, a comprehensive literature review found that evidence is limited due to methodological issues and results are mixed [18, 25]; thus, the relationship may not be as strong as generally thought. Researchers suggest more qualitative research to explore how pregnancy is conceptualized in different social and cultural arenas [18].

As it is currently operationalized, unintended pregnancy occurs at higher rates among low-income women and women of color than other populations [22]. Their pregnancies have thus been the focus of research and targeted for prevention, attention that could be interpreted as problematizing pregnancies among these women. From a health equity lens, it is useful to further examine if intentions-based measures disproportionately mislabel poor women of color’s experiences of getting pregnant as being unintended and causally linked to poor health outcomes. Examining these issues among a diverse sample of women and men, of various educational, racial/ethnic, and economic backgrounds, can facilitate creating a unified framework of pregnancy that reflects the lived experiences of people of a wide range of backgrounds.

Because of the vast amount of resources devoted to reducing unintended pregnancy and the negative framing of pregnancy for certain groups, valid measurement of the construct is essential. By improving our understanding of this construct, researchers should be better able to measure the phenomenon of becoming pregnant. Moreover, researchers and clinicians alike can avoid misclassifying individuals who do not articulate prospective intentions or planning regarding childbearing, as well as focus on the conditions under which people get pregnant, and develop more appropriate interventions to assist with either avoiding or supporting pregnancy.

The phenomenon of ‘getting pregnant,’ as we refer to here, is a construct that deserves further exploration free of the assumptions associated with the pregnancy intentions framework. An in-depth understanding of how women and men, people of diverse socioeconomic positions and racial/ethnic backgrounds, with and without children conceptualize pregnancy, *in their own words*, is necessary to improve measurement, health services, and better direct public health resources.

## Methods

### Sampling & Data Collection

The Social Position and Family Formation (SPAFF) study comprises 200 semi-structured interviews from a community–based sample of heterosexual women and men (ages 18–35), of diverse backgrounds, with and without children. The broad aim of this parent study was to examine how factors related to social position influence family formation decisions. Individuals were sampled from select neighborhoods in New York City and northern New Jersey to reflect the racial/ethnic, educational, and income distribution of the larger metropolitan area and were recruited from public venues such as cafes and laundromats. A purposive sampling approach sought to recruit participants who were: aged 18 to 35 years, living in one of the selected neighborhoods, and whose primary language was English or Spanish. Given the sampling goal of including individuals of diverse backgrounds, recruitment by sex, income, and relationship status was monitored to ensure broad distribution of those characteristics in the final sample. Recruitment procedures and sampling approach are described in greater detail elsewhere [26].

Screening for eligibility was followed by informed consent; in-depth interviews (IDIs) were conducted by a team of extensively trained interviewers. A semi-structured interview guide explored various domains related to family formation decision-making. With regard to our interest in understanding how people specifically view becoming pregnant and childbearing, relevant questions included “What do you think is a good age and situation for having a child?” and “Do you two [you and your partner] discuss the possibility of having children? What are these conversations like?”

Participants were given $50 for their participation in the study ($5 gift card for screening; $45 for IDI). Interviews lasted approximately 1 hour, were audio-recorded, and professionally transcribed. This study was approved by the City University of New York (CUNY) Institutional Review Board (Protocol #337386–2).

### Analysis

We used a grounded theory approach for the analysis by first allowing for the inductive development of the code structure directly from the data (transcripts). Initial analytic codes focused on all descriptions related to pregnancy and childbearing and subsequent line-by-line coding identified repeating ideas in the data [27, 28]. Using a consensus-based iterative approach, four analysts collectively established a coding structure and definitions. The research team coded repeating ideas related to how pregnancy ‘happens’ in the participants’ own words, while concurrently generating descriptive and interpretive memos. All transcripts were coded and reviewed by the research team in several rounds. As new codes were applied to subsequently reviewed transcripts, previous transcripts were reviewed and updated according to the final coding structure. Together the analytic team grouped codes and repeating ideas focused on salient themes comprising a possible theoretical construct regarding the notions individuals hold related to pregnancy [27].

From the coding process emerged a pattern of notions regarding pregnancy that could be categorized in the following way: 1) *deliberate,* 2) *predetermined,* or 3) *blend of both*. Each interview was assigned to one of these three main categories of the emerging ‘getting pregnant’ construct. The analytic team consulted with one another regarding cases in which category determination was unclear, after which 24 cases for whom a determination could not be definitively made were excluded; these participants had not discussed pregnancy in sufficient detail.

Because analysis also revealed that participants talked about future and past pregnancies differently, we applied relevant coding to all transcripts to perform a thematic sub-analysis.

The mixed-methods analytic software Dedoose (V7.6.24) was used for all qualitative analyses. Data collection and analysis were informed by the standards for assessing qualitative research quality [29]. The quantitative software R (V3.4.2) was used to obtain descriptive statistics and perform bivariate analyses of the demographic characteristics of the sample. Participants’ names are pseudonyms largely chosen by the participants themselves.

### Availability of data and materials

The dataset supporting the conclusions of this article is not publicly available. De-identified data are available upon request.

## Results

We present the sociodemographic characteristics of the total sample, followed by the qualitative thematic findings and sample characteristics within each category of the ‘getting pregnant’ construct that emerged from the analysis.

### Sample description

Of the 200 interviewees, 176 who articulated notions about getting pregnant are included in this analysis. Of those, 47% were male and 53% female, with a mean age of 27.7 years (Table 1). Over half (62%) did not have children. In terms of relationship status, the highest proportion was single (40%), followed by married (21%). Almost half (48%) of participants had a Bachelor’s degree or above; half (50%) had an annual income of $20,000–$59,999. More than a third (36%) were African-American or Black, followed by 31% Hispanic, and 26% White.

**Table 1:** Sample characteristics

Characteristic	Total (*N* = 176) n (%)	Deliberate Only (*n* = 73) n (%)	Predetermined Only (*n* = 9) n (%)	Blend (*n* = 94) n (%)
Age				
Mean years (SD)	27.7 (4.6)	27.9 (4.8)	26.8 (4.5)	27.5 (4.6)
Sex**				
Male	82 (47)	44 (60)	3 (33)	35 (37)
Female	94 (53)	29 (40)	6 (67)	59 (63)
Children***				
Yes	67 (38)	14 (19)	4 (44)	49 (52)
No	109 (62)	59 (81)	5 (56)	45 (48)
Relationship				
Single	70 (40)	28 (38)	5 (56)	37 (40)
In a Committed Relationship	28 (16)	13 (18)	0 (0)	15 (16)
Living together	29 (17)	14 (19)	4 (44)	11 (12)
Divorced/Separated	8 (5)	3 (4)	0 (0)	5 (5)
Married	37 (21)	15 (21)	0 (0)	22 (23)
In Open Relationship	4 (2)	0 (0)	0 (0)	4 (4)
Education				
< High school (HS) completion	6 (4)	1 (1)	1 (11)	4 (4)
HS diploma or GED	18 (11)	5 (7)	0 (0)	13 (14)
Some college/technical school or Associates	66 (38)	25 (35)	4 (44)	37 (40)
Bachelor’s or above	82 (48)	40 (56)	4 (44)	38 (41)
Income				
$0–$19,999	41 (23)	14 (19)	1 (11)	26 (28)
$20,000–$59,999	87 (50)	34 (47)	7 (78)	46 (49)
≥ $60,000	47 (27)	25 (34)	1 (11)	21 (23)
Race/Ethnicity*				
African-American/Black	63 (36)	19 (26)	4 (44)	40 (43)
White	46 (26)	27 (37)	2 (22)	17 (18)
Hispanic	55 (31)	20 (27)	2 (22)	33 (35)
Asian/Pacific Islander	10 (6)	5 (7)	1 (11)	4 (4)
Other	2 (1)	2 (3)	0 (0)	0 (0)

*p < 0.05; **p < 0.01; ***p < 0.001

### Categories of the “getting pregnant” construct

**Fig. 1:**
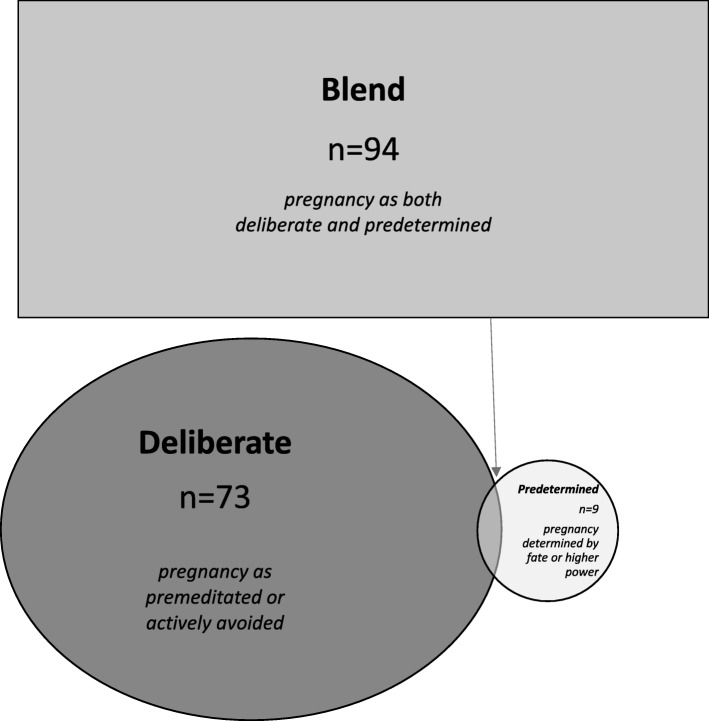
Notions of Pregnancy

The ways in which participants spoke about getting pregnant, or childbearing, revealed a construct consisting of three notions categorized as deliberate, predetermined, or blended (Fig. 1). One group of participants used language that solely described pregnancy as a deliberate process that was either premeditated or actively avoided. The second group described pregnancy as a predetermined phenomenon that came about due to fate or a higher power and was essentially out of one’s control. The third group of participants represented a blending of both notions, talking about pregnancy as something that could be both deliberate and predetermined. Below we describe each category of this construct in detail.

### Deliberate notions of pregnancy

Approximately 41% of the sample (*n* = 73) expressed only deliberate notions related to the construct of ‘getting pregnant.’ Pregnancy was described as a mindful choice, where it was planned to happen at a certain time. This type of premeditated decision-making was often mentioned with reference to communication with a partner. Discussions of having children focused on optimal timing in terms of, for example, age of parents or siblings, or under certain circumstances such as financial stability. One woman in a committed relationship described how she thought about the timing of having children:

“Maybe I’ll have kids…like at 33…once I have everything settled….I wouldn’t like want to rush, kind of jump into having kids, and how many would I be able to have, and what kind of years would I want in between them….‘Cause I do want, I mean, at least two, and I would definitely be open, if having kids myself doesn’t work out like to adopt kids too. So it’s a lot of timing. It’s a lot of pressure with timing when you’re, like getting into your thirties.” [Age 30, White, Female, No Children]

Most of the participants who used deliberate-only language did not have children and highlighted the lifestyle changes and financial commitment associated with having children. In that vein, many participants noted not wanting to have children in the near future.

Max, who was living with his significant other, talked about the ideal circumstances under which he would like to have a child.

“Both of us work and I want us to be comfortable ….We want to make sure we can kind of set everything up and be proactive about bringing in a family.” [Age 26, White, Male, No Children]

For participants expressing deliberate notions only, financial stability was discussed as a key factor in decisions related to pregnancy and family formation. These discussions reflected their personal situations, but some projected that others should also aim to deliberately plan to have children only after financial stability has been achieved. Amelie, who was single and did not think she wanted to have children because of the time and energy investment, described her frustration with others who have children without fully being able to financially support them:

“It boggles my mind and I just don’t understand why people have kids who can’t afford to have kids. And I see that and I don’t want to do that….And I think that everything is a decision.” [Age 27, White, Female, No Children]

### Predetermined notions of pregnancy

Only nine participants (5%) exclusively described pregnancy as a phenomenon that was predetermined by fate or a higher power, about equally divided between those with and without children. This notion encompassed two sub-categories that we termed ‘naturalistic’ and ‘chance.’ Naturalistic language refers to descriptions of pregnancy as something that is an organic, natural process, not one that can be scheduled. Richard, a single man who had lived in the United States for only a few years since emigrating from the Netherlands explained his position on family formation:

“It’s definitely not something you can entirely plan, though, having a family….You cannot tell yourself, ‘Look, I will not start a family until I have paid off student loans,’ that’s not how those things work, and I feel like it’s very much an organic thing.” [Age 24, White, Male, No Children]

Others spoke about pregnancy using similar language that suggested it was something predetermined, but without descriptions of it being a natural process. This group fell into the ‘chance’ sub-category. Many participants described pregnancy as something that ‘just happens,’ in the past or in the future. Keisha, who lived with her boyfriend and had had a miscarriage conveyed this perspective succinctly:

“…if it happens, it happens. That’s it.” [Age 26, African-American/Black, Female, No children]

The other terminology around chance was in opposition to the deliberate perspective, specifically using negative qualifiers of deliberate verbs as in one *doesn’t* choose, and *can’t* predict. Renee, who was recently single after a long-term relationship, said about having children:

“I didn’t want three [children], but God gave me three. I didn’t want none, but they came.” [Age 32, African-American/Black, Female, Has children]

### Blended notions of pregnancy: both deliberate and predetermined

Just over half (53%) of participants used some combination, or blend, of deliberate and predetermined language as it relates to pregnancy, about equally divided between those with and without children. These statements sometimes seemed contradictory. Charlotte, who was married and said she could not imagine having more children given the size of their small New York City apartment, used blended language to suggest pregnancy can be planned, but also was not completely within her control.

“…to a certain extent you plan. But there’s only so much you can control with that either. I suppose we will plan. Reach a point in which we say, ‘Oh. I think I’m ready to try again and have another baby.’ Then let things happen as they happen.” [Age 30, White, Female, Has Children]

Initially Ashley, who was living with her male partner, talked about getting pregnant using language that alluded to deliberate planning, but then later discussed wanting more children, using predetermined language:

“…you know I’m not planning to have another one because he adds up, that’s just more money….I do want a bigger family, just not right now. God will give it to me I just don’t know when. That’s all.” [Age 29, Hispanic, Female, Has Children]

In both Charlotte’s and Ashley’s blended descriptions, they imply that there are multiple moments in the process of getting pregnant, with each moment potentially being steered by different perceptions of how pregnancy happens. For Charlotte, planning is sequenced first, followed by a passive action happening second. Whereas Ashley describes planning not to get pregnant overlapping in time with the possibility that a pregnancy will happen.

### Demographic differences in conceptualization types

Those in the deliberate group were more likely to be male (*p* < 0.01), not have children (*p* < 0.001), and be White (*p* < .05; Table 1). By contrast, those who articulated a predetermined or blended notion of pregnancy were equally likely to have children or be childless, and more likely to be female (*p* < 0.01) and African-American/Black (*p* < 0.05).

### Past versus future pregnancies

**Fig. 2:**
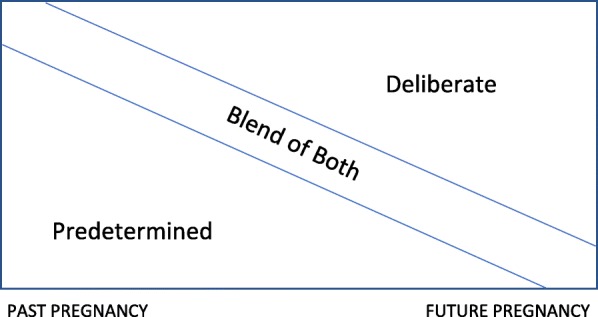
Notions of Past versus Future Pregnancies for those who had prior pregnancy/children

Analyses revealed a distinction in how participants discussed past versus future pregnancies. General comments of pregnancies or having children, without specific reference to the interviewees themselves, were coded as participants’ thoughts about *future* pregnancies. Deliberate-only language was predominantly used to describe future pregnancies (Fig. 2). In contrast, participants who discussed a past pregnancy often did so with predetermined-only notions. (Blended language was also used to describe both future and past pregnancies.) John, a self-described “family guy” talked about the first time a partner became pregnant:

“We got involved and unexpectedly, she got pregnant…Honestly, I didn’t want to have a child at that age….It just happened.”

Later in the interview, he discusses his thoughts about having future children, using deliberate language:

“…It always comes back to finances, so financially, I don’t think it’s good for us to have any more children. There’s going to be no more.” [Age 34, African-American/Black, Male, Has Children]

## Discussion

These findings provide insight into how young women and men conceptualize the phenomenon of getting pregnant. The deliberate notions many participants held aligns with the commonly used concepts of intentions, planning, and decision-making as they relate to pregnancy. The finding that men in the sample held deliberate notions more than women suggests that they may perceive greater control over pregnancy. That most people who held deliberate notions of pregnancy were without children may be reflective of their ‘successful’ efforts to prevent pregnancy to date, thus reinforcing their own sense of ability to actively pursue and/or control fertility. In contrast, those with children may have a more nuanced understanding of the web of context, events, and feelings or emotions surrounding the experience of becoming pregnant.

Predetermined notions, particularly among those who used chance terminology, were often presented relative to and as the ‘negative’ opposite of deliberate language. This finding highlights the societal dominance of the deliberate (i.e., intentions) perspective around pregnancy and the lack of a broader vocabulary to more fully articulate the predetermined perspective. That deliberate language is embedded in long-standing conceptualizations and measures of pregnancy may have impeded a more expansive and complete representation of individuals’ notions about becoming pregnant. In either case, findings suggest a need to expand the current paradigm and operationalization of the ‘getting pregnant’ construct.

Although related, the term predetermined is distinct from what is known in the literature as fatalistic, given that participants who employed this notion did not discuss the inevitability of pregnancy (nor with particularly negative connotations) so much as it being by chance or through naturalistic means. Of note, the proportion of participants who only used predetermined language was small (5%) and the highest proportion was among those who held blended beliefs (53%), suggesting that views of pregnancy are more complex than our current conceptualization as either fatalistic or planned [13, 30, 31]. The finding that participants of a range of income, educational, and racial backgrounds held both deliberate and predetermined notions (blend category) of pregnancy demonstrates people’s tendency to use either competing perspectives to conceptualize the experience of pregnancy or that getting pregnant is an experience made up of micro-moments that may each be characterized in distinct and sometimes incongruous ways. This could be a manifestation of an internal conflict in that individuals believe they can and should plan pregnancies, but may leave pregnancy to chance or fate because of the responsibilities associated with childbearing in the larger context of their lives [19]. Another possibility is that individuals view procreation as a fundamental aspect of the human condition that defies complete control or planning [13, 30, 32]. Our findings support a more robust and complex construct, as compared to the prevailing intentions framework, including notions that pregnancy can be predetermined and/or something that ‘just happens.’ This builds on other research that has found that low-income women do not always articulate pregnancy intentions and describe pregnancy as something that may “just happen.” [13]

Differences in how people discuss past and future pregnancies suggest that they may be more inclined to attributing past pregnancies as occurring in a way that was predetermined and not actively decided upon. By contrast, future pregnancies were mainly described using deliberate notions, suggesting that, in the context of the future, there is greater tendency toward the current societal framework of *planning* pregnancies. Thus, predetermined and deliberate notions regarding pregnancy may coexist in the same individuals, while also varying in reference to the past or future. This finding likely relates to why reports of pregnancy intention based on timing of data collection (retrospective, current, and prospective measurement) may differ [2, 33].

Participants in all groups spoke not only about pregnancy, but also about notions related to family formation and parenting. Further exploration is needed to understand if and how distinct notions of getting pregnant are reflective of differences in how people may think about pregnancy itself, as distinct from having a child. This may explain some of the variability in capturing pregnancy intentions, in that some may view intentions as it relates to pregnancy, whereas others associate intentions with family formation and parenting.

Historically, researchers have conceptualized and measured pregnancy and family formation through an intentions-based framework, whether explicitly [2, 34–36] or implicitly [15, 22]. Over the last several decades, our understanding of human behavior has evolved to recognize that behavior is not always motivated by rational thinking [37, 38]. For example, in data from 2011 to 2013, 10% of women at risk of unintended pregnancy had reported not currently using contraception [39]. That sexual activity continues in the absence of contraception (among those who report not wanting to become pregnant) supports the notion that there is more to consider beyond the ‘risk’ of an unplanned pregnancy. This phenomenon has heretofore been referred to and conceptualized as ‘irrational’ behavior. In light of our findings, we clearly need enhanced language to more accurately capture the different ways in which people conceptualize getting pregnant. Recent suggestions have been to assess measures of reproductive autonomy as the desired outcome of interest, as opposed to intentions [40], or pregnancy “supportability,” that is, the extent to which a woman’s pregnancy is supported by her health, partner, family, health care team, and more structural social and health care policies [41].

The results should be interpreted within the limitations of the study. Our sample was drawn from a large, urban setting and would need to be explored among those in non-urban areas, for transferability of findings. Questions incorporated deliberate-like language, which may have encouraged responses in kind. Thus, the findings may over-represent conceptualization of ‘getting pregnant’ as deliberate. We were not always able to analyze reasons for or context surrounding participants’ use of certain language related to pregnancy, due to a lack of discussion and probing. Participants were also interviewed at different points in their lives in terms of family formation, thus, their perspectives may reflect their current situation in their life course.

Our findings have implications for the health care setting and future measurement of pregnancy intentions. In clinical encounters, individuals may characterize the potential of a future pregnancy with deliberate language, yet may also believe pregnancy is a predetermined phenomenon. As such, given the difficulty in predicting pregnancy in light of the complexity of the construct, providers might consider asking patients directly if there are any reproductive health services they desire instead of relying solely on responses to yes/no questions about pregnancy intentions that are being increasingly promoted [3, 4, 42, 43].

## Conclusions

Our findings facilitate a unified framework, of women and men of a range of socioeconomic and racial/ethnic backgrounds, that encompasses distinctions between and a blending of various notions of getting pregnant. This framework offers a holistic conceptualization (with new terminology) that more closely aligns with the lived experiences of pregnancy and can be operationalized and tested as a measurement tool as a next step. New pregnancy-related measures should be developed to improve on old frameworks that presume the universality of ‘rational’ decision-making and planning in pregnancy. Instead, our findings support expanding measures to incorporate the notion of pregnancy as being both a deliberate event and one that may simultaneously be predetermined. Future work can explore why and when pregnancy is understood simultaneously as a deliberate and predetermined event; what distinguishes those who hold discrete notions of pregnancy; and how the construct of ‘getting pregnant’ can be measured in a meaningful way that aligns with the perspectives of women and men. These results expand our understanding of individuals’ notions about getting pregnant and can guide more valid approaches to measuring this construct going forward. Refined measurement can help direct public health funding and develop more appropriate interventions.

## Data Availability

The datasets used and/or analyzed during the current study are available from the corresponding author on reasonable request.
